# The American System of Dentistry

**Published:** 1887-04

**Authors:** 


### Bibliographical.

---

THE AMERICAN SYSTEM OF DENTISTRY.—In treatises by various authors. Edited by Wilbur F. Litch, M. D., D. D. S. Volume II, Operative and Prosthetic Dentistry, with one thousand and thirty-five illustrations. Publishers: Lea Brothers & Co., Philadelphia, Pa., 1887.

The second volume of this excellent work has been published in the same superior style as the first, and to many it will prove to be more interesting on account of its practical treatises.

The contributors and subjects are "The Stopping Process with Gold, and the Relative Procedures," by Louis Jack, D. D. S. "The Herbst Method of Filling Teeth," by C. F. W. Bœdecker, D. D. S. "Plastic Materials for Filling Teeth," by A. G. Bennett, D. D. S. "Electro-Chemical Relations of Stoppings to the Teeth," by S. B. Palmer, D. D. S. "Calcareous Deposits on the Teeth," by A. W. Harlan, M. D., D. D. S. "Discolored Teeth and their Treatment," by James Truman, D. D. S. "Orthodontia," by S. H. Guilford, D. D. S. "Replantation and Transplantation of the Teeth," by George W. Weld, M. D., D. D. S. "Extraction of Teeth," by Thos. C. Stellwagen, M. D., D. D. S. "Taking Impressions of the Mouth," "Plaster Models," "Antagonizing and Contour Models," by A. G. Bennett, D. D. S. "Articulation of the Human Teeth," "The Anatomical Articulator," by W. G. A. Bonwill, D. D. S. "Metallic Dies and Counter-Dies," by William H. Truman, D. D. S. "Artificial Dentures on Bases of Gold and Silver," by William H. Truman, D. D. S. "Continuous Gum Work," by D. D. Smith, M. D., D. D. S. "Artificial Dentures on Bases of Fusible Alloys," by Theodore F. Chupein, D. D. S. "Artificial Dentures on Rubber Base," by A. P. Beale, D. D. S. "Celluloid and Zylonite," by W. W. Evans, M. D., D. D. S. "Artificial Crowns," (Pivot Teeth), by William H. Truman, D. D. S. "A System of All-Porcelain Crown Substitution," by W. G. A. Bonwill, D. D. S. "Crown-and-Bridge-Work," by Wilbur F. Litch, M. D., D. D. S. "Metallic Facings for Carious Crowns," by Wilbur F. Litch, M. D., D. D. S. "Moulding and Carving Porcelain Teeth," by William R. Hall, D. D. S. "The Hygienic Relations of Artificial Dentures," by Edw. C. Kirk, D. D. S. "Dental and Facial Types," by Robt. S. Ivy, D. D. S. "Obturators and Artificial Vela," by Henry A. Baker, D. D. S. Among other interesting articles the Third Volume will contain one on Oral Surgery by Prof. L. McLane Tiffany, Professor of Oral Surgery in the University of Maryland Dental Department.

The third volume will complete the "System," and all will comprise a work of great value to the dental library of every practicing dentist.

